# Novel high-efficient adsorbent based on modified gelatin/montmorillonite nanocomposite for removal of malachite green dye

**DOI:** 10.1038/s41598-024-51321-2

**Published:** 2024-01-12

**Authors:** Mahmoud H. Abu Elella, Nema Aamer, Heba M. Abdallah, Eduardo A. López-Maldonado, Yasser M. A. Mohamed, Hossam A. El Nazer, Riham R. Mohamed

**Affiliations:** 1 Chemistry Department, Faculty of Science, Cairo University, Giza, 12613 Egypt; 2 Polymers and Pigments Department, Chemical Industries Research Institute, National Research Centre, Dokki , Giza 12622 Egypt; 3 Faculty of Chemical Sciences and Engineering, Autonomous University of Baja California, CP: 22390 Tijuana, Baja California Mexico; 4 Photochemistry Department, National Research Center, Dokki, Giza 12622 Egypt

**Keywords:** Polymer chemistry, Pollution remediation, Pollution remediation

## Abstract

Shortage of drinking water has gained potential interest over the last few decades. Discharged industrial effluent, including various toxic pollutants, to water surfaces is one of the most serious environmental issues. The adsorption technique has become a widely studied method for the removal of toxic pollutants, specifically synthetic dyes, from wastewater due to its cost-effectiveness, high selectivity, and ease of operation. In this study, a novel gelatin-crosslinked-poly(acrylamide-*co*-itaconic acid)/montmorillonite (MMT) nanoclay nanocomposites-based adsorbent has been prepared for removing malachite green (MG) dye from an aqueous solution. Modified gelatin nanocomposites were synthesized using a free-radical polymerization technique in the presence and absence of MMT. Various analytical instrumentation: including FTIR, FESEM, XRD, and TEM techniques were used to elucidate the chemical structure and surface morphology of the prepared samples. Using a batch adsorption experiment, Langmuir isotherm model showed that the prepared modified gelatin nanocomposite had a maximum adsorption capacity of 950.5 mg/g using 350 mg/L of MG dye at pH 9 within 45 min. Furthermore, the regeneration study showed good recyclability for the obtained nanocomposite through four consecutive reusable cycles. Therefore, the fabricated gelatin nanocomposite is an attractive adsorbent for MG dye elimination from aqueous solutions.

## Introduction

Water is a fundamental concern for human life and ecosystem as well. Recently, notably, fast-paced industrialization and growing population have attracted more attention due to the discharge of toxic effluents onto water surfaces^1,2^. Synthetic organic dyes are considered one of the most significant pollutants that are utilized in several industrial areas, for instance, textiles, printing, etc. They cause a large amount of contamination in our environment. Cationic dyes are non-biodegradable, the primary source of contaminated water, and not easily eliminated using conventional removal approaches^3–7^. For example, triphenylmethane dyes are shining, deeply colored, and based on the hydrocarbon triphenylmethane in their molecular structure, such as Malachite green (MG) dye. MG is a cationic organic dye and is widely applied in several industries, including food, paper, textiles, leather, printing, and cosmetics^8^. However, it has outstanding disadvantages. For example, it produces stable, non-biodegradable, and carcinogenic byproducts that cause a variety of human health problems, including carcinogenicity, eye irritation, mutagenesis, headache, and malformation^9,10^.

To solve this environmental problem, various conventional techniques can be applied for the purification of dye-based contaminated water, including electrochemical^11^, catalytic degradation^12^, membrane separation^13^, photo-degradation^14^, and coagulation-flocculation^14^. However, there are various essential disadvantages, including high-cost techniques, long-time and high-energy consumptions, and low-separation capabilities. In the past decades, adsorption approach has played a crucial role in water purification applications to remove toxic dyes thanks to its remarkable advantages, for instance being a cost-effective method, as well as its high removal efficiency, low operating costs, and high performance^15,16^.

So, designing adsorbents with excellent separation efficiency is an attractive strategy. As a result, over the past few years, biodegradable polymer nanocomposite had great attention in the adsorption approach to purify polluted water from pathogenic effluents. Biodegradable polymeric materials, including gelatin, chitosan, etc. are being widely applied to eliminate wastewater pollutants. Abu Elella et al*.* is very interested in developing high-efficient adsorbents based on low-cost biodegradable and biocompatible materials for the removal of different pollutants from an aqueous solution. For the removal of cationic dyes, our research group had published several researches in literature^17–22^ based on modified natural polymers.

Gelatin is considered an attractive natural biodegradable polymeric material. It is a hydrolysis product extracted form collagen with a structural formula (NH_2_COOH–CH–R), where R is an amino acid derived from glycine, proline, or hydroxyproline^23^. Gelatin has several outstanding advantages, including biocompatibility, non-toxicity, low-cost material, biodegradability, and availability. It has several attractive functional moieties: amino, carboxyl, and hydroxyl moieties, which make it an effective adsorbent^24,25^.

Although Gelatin has some drawbacks: weak thermal stability, low removal separation, and fast degradability in water, which made it lose its capability to be applied as a suitable adsorbent for wastewater treatment without any modification^12,13^.

Natural polymer nanocomposites are intensively used for removing various dyes from contaminated water due to their unique properties like; fast kinetics, recyclability, good adsorption performance, and low cost^26^. Among them, gelatin-based nanocomposites exhibit better physicochemical characteristics than unmodified gelatin, such as good thermal stability, excellent separation efficiency, and good recyclability^27^. In the designing of gelatin nanocomposites, numerous different nano-fillers have been employed, among them, multilayer nanoclays. These materials, which have clearly defined and manageable morphologies with appropriate sizes and porosities, are potential adsorbents for treating wastewater due to their outstanding features including high surface area, significant chemical reactivity, mechanical properties, cost-effectiveness, specific selectivity, sustainability, recyclability, low power consumption, and the ease of chemical or physical modification^28–30^. Natural polymer nanocomposites based on Montmorillonite (MMT) nanoclay, have been widely used for adsorbing different pigments and dyes from contaminated water^31–33^. MMT is a smectite-family phyllosilicate mineral composed of two silica tetrahedral sheets jammed together with one edge-shared octahedral sheet of aluminum hydroxide. Its chemical formula is hydrated sodium calcium aluminum magnesium silicate hydroxide, (Na, Ca) × (Al Mg)_2_(Si_4_O_10_)(OH)_2_⋅nH_2_O^34^. Its high surface area is due to its layered structure, which has an excellent cation exchange capacity, making it suitable for organic pigments and dyes removal from water*.* The nano-structured montmorillonite clay enables it to be applied in three-dimensional cross-linked composites, which are used for dye adsorption due to their high adsorption capacity, stability, and good thermal stability^21,35^.

The current study intends to synthesize a highly efficient modified gelatin nanocomposite as an adsorbent for the capture of toxic cationic organic dye (malachite green) from an aqueous solution via an adsorption batch approach. The gelatin-grafted-poly (acrylamide (AAM)-*co*-itaconic acid (IA))/MMT nanocomposite is prepared via free radical polymerization technique using crosslinking agent *N, N*-methylene bisacrylamide. Furthermore, the prepared gelatin nanocomposites’ structure was elucidated using various physicochemical analysis techniques such as FE-SEM, FTIR, EDX, XRD, and TEM while the thermal stability was investigated via TGA. The adsorption study of the synthesized nanocomposites was studied in aqueous solution nanocomposites under the effect of various factors. The findings confirm that the fabricated nanocomposite adsorbent showed remarkable separation for MG dye with excellent adsorption capacity, which will promise a low-cost and effective adsorbent for the wastewater treatment field.

## Experiments

### Materials

Gelatin was obtained from S.D. Fine Chemical, India, and Ammonium persulfate (APS) was bought from Maharashtra, India, and IA and MBA were purchased from Sigma-Aldrich, Germany. AAM and MG were purchased from Loba-Chemi Pvt. Ltd., India. Also, Hydrochloric acid and sodium hydroxide were purchased by Merck-Germany. Nanoclay, Nanomer 1.31PS, montmorillonite nanoclay was provided from Aldrich, Germany.

### Experimental methods

#### Synthesis of grafted gelatin/MMT nanoclay nanocomposites

The gelatin-*cl*-poly (AAM-*co*-IA)/MMT nanoclay nanocomposites were synthesized by in-situ homogenous dispersion of different MMT nanoclay concentrations (1–5%) (w/w) via free radical grafting polymerization technique using crosslinking agent *N, N*-methylene bisacrylamide. Initially, 1.0 g of gelatin was dissolved in 25 mL of distilled water for 20 min under constant stirring. After that, different concentrations of MMT nanoclay suspension (1, 2, 3, 4, and 5% of the total gelatin weight) were dispersed homogenously in the abovementioned solution for 30 min under constant stirring without disturbance. Subsequently, 25 mM APS was dissolved in 5 mL distilled water and then added to gelatin solution at 60 °C under 15 min stirring by purging N_2_ gas. Following both acrylamide (0.3 M) and itaconic acid (0.3 M) were added to the gelatin/MMT mixture. Next 5 wt.% of MBA solution was partially poured under stirring at 60 °C. After 2.5 h, the obtained nanocomposites were purified using ethanol/ distilled water solution under stirring at 50 °C, and then washed with distilled water several times to remove any unreacted materials. Finally, the purified nanocomposites were dried at 50 °C for 48 h. The controlled grafted gelatin hydrogel using 5 wt.% of MBA was synthesized according to the above pathway in the absence of MMT nanoclay as a control sample.

#### Adsorption batch studies

The adsorption of MG dye was performed using gelatin-*cl*-poly (AAM-*co*-IA)/MMT nanocomposites under the effect of various parameters like; MMT nanoclay concentration, MG initial concentration, pH of MG solution, weight of adsorbent, and contact adsorption time. 10 mg of polymer was immersed into MG (10 mL) at room temperature (30 °C) for 60 min in the range of pH (2–9). The dyed samples were decanted, and then the residual MG concentration was determined using a UV–Vis spectrophotometer at a wavelength of 670 nm.

Equation (1) was used to calculate the adsorption capacity of MG at equilibrium.

1 $$\mathrm{Adsorption \,\,capacity }({\text{Q}}) =\frac{(C0-Ce)}{W}\times V$$

where C_o_ and C_e_ are the initial and equilibrium MG concentrations (mg/L), respectively. V is the soaked volume of MG solution (L), and W is the gelatin montmorillonite nanocomposites weight (g).

#### Regeneration study

The regeneration (adsorption–desorption) ability of the modified gelatin/MMT nanocomposite was carried out within 4 consecutive cycles. The reusability test occurred via soaking MG-dyed nanocomposite sample in 100 mM HCl (desorbing agent). After 24 h, the non-dyed nanocomposites were extracted with decantation and then washed and neutralized by 100 mM NaOH, and subsequently dried in a vacuum oven at 50 °C. Desorption of MG % was determined using the following Eq. (2)^36^.

2 $$\% \, \mathrm{MG \,\, desorption }=\frac{\mathrm{Desorbed  \,\,conc}.({\text{mg}}/{\text{L}})}{\mathrm{Adsorbed \,\, conc}. ({\text{mg}}/{\text{L}})}\times 100$$

## Characterization

The chemical structure of the prepared gelatin-*cl*-poly(AAM-*co*-IA)/MMT nanocomposites was elucidated, comparing with non-modified gelatin, MMT, and gelatin hydrogel, via various analytical instrumentation such as:

- FTIR (Jasco 4100, Japan) was used to determine the chemical structure within the wavenumber 4000–400 cm^−1^ at 25 °C.
- XRD, a Philips Xpert MPD Pro, is used at 50 kV, 40 mA, 3°/s as a speed scan rate to demonstrate the crystallinity of the prepared nanocomposites.
- Shimadzu Thermogravimetric Analyzer was used to determine the thermal stability of the prepared nanocomposites (TGA-50H). The temperature range was 25 to 800 °C with a heating rate of 10 °C/min in a nitrogen atmosphere.
- Surface morphology for tested samples was investigated with FE-SEM (Quanta 250) at various magnifications and 30 kV. The FE-SEM technique is equipped with an EDX unit to investigate all incorporated elements for gelatin nanocomposite.
- TEM images of polymer nanocomposites compared with MMT nanoclay were taken using a JEM-100S Transmission Electron Microscope (TEM, Japan).
- MG adsorption studies were performed with a Unico 1200 UV–Vis spectrophotometer set to max wavelength = 670 nm.

## Results & discussion

### Preparation of modified gelatin/ MMT nanocomposites

**Figure 1:**
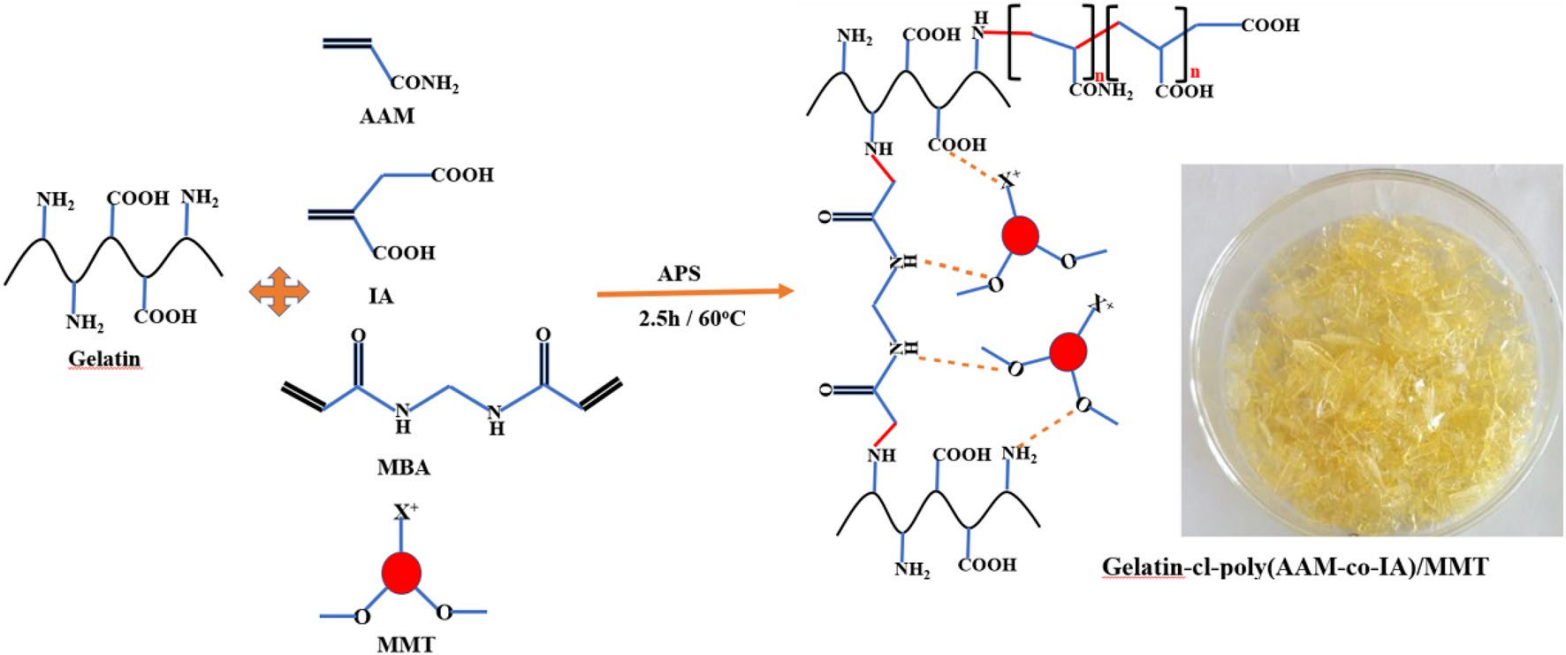
Schematic illustration of the preparation of gelatin-cl-poly (AAM-co-IA)/MMT nanocomposites via free radical polymerization technique.

Gelatin-*cl*-poly(AAM-*co*-IA)/MMT nanocomposites were synthesized via free-radical polymerization method using APS and MBA as an initiator, and crosslinking agent, respectively, and MMT as a nano-filler (Fig. 1). According to Fig. 1, APS produces sulfate anion radicals by heating which attract hydrogen atoms from –NH_2_ groups on gelatin backbone, then acrylamide and itaconic acid were grafted onto gelatin side chains. MBA acts as a cross-linker by coupling the end vinyl groups in MBA molecules with the free NH radical of gelatin. MMT nanoparticles were in situ dispersed within copolymer chains to form the 3D structure of the hydrogel nanocomposite.

### Characterization of modified gelatin/ montmorillonite nanocomposite

**Figure 2:**
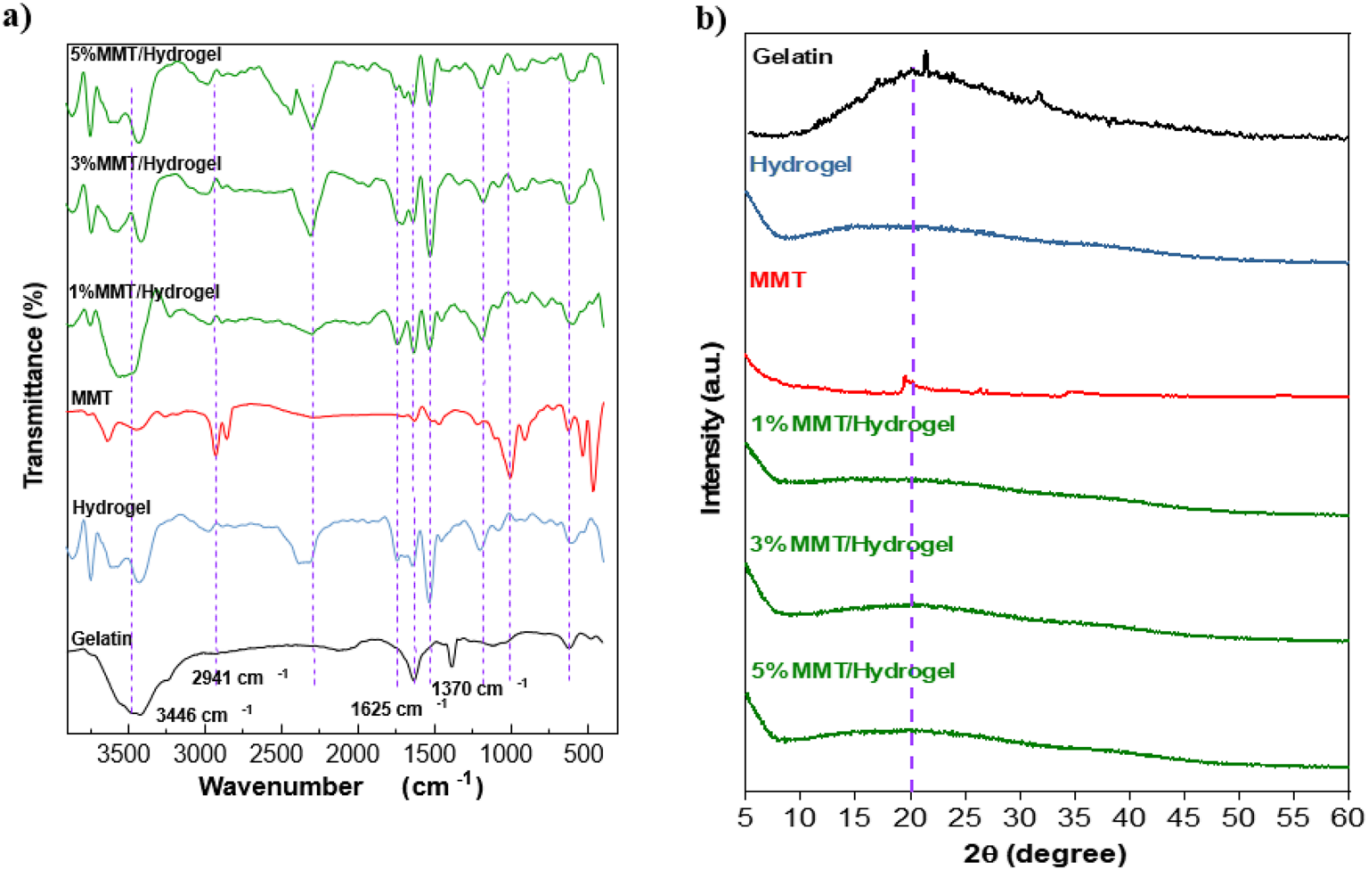
(**a**) FTIR spectrums and (**b**) XDR patterns for unmodified gelatin, hydrogel, MMT, and 1% to 5% MMT/hydrogel nanocomposites.

Various analysis techniques were performed to characterize the structure of the prepared gelatin-*cl*-poly (AAM-*co*-IA)/MMT nanocomposites compared to native gelatin, MMT, and gelatin hydrogel in absence of MMT. Figure 2a depicts the FTIR spectra of MMT, non-modified gelatin, gelatin-*cl*-poly (AAM-*co*-IA)/hydrogel and modified gelatin/MMT nanocomposites containing (1, 3, and 5 w/w%) of MMT.

The FTIR spectrum of non-modified gelatin shows the characteristic signals of the polypeptide. In the region of 3584 cm^−1^ to 3407 cm^−1^, a broad band is observed, which is associated with the narrowing of the –OH and –N–H bonds for the secondary amides^37^. The presence of the amide A band at a higher wave number is associated with less degradation of the gelatin chains and a high molecular weight structure predominates^38,39^. The asymmetric stretching vibration band of =C–H and ammonium correspond to the peak of type B amide. The low intensity band at 2941 cm^−1^ is attributed to the symmetric and asymmetric vibrations of the –CH_2_ group^40^. It has been reported, the tendency of the stretching vibrations of amide A, amide B and –CH_2_ to overlap due to possible dimeric intermolecular associations of carboxyl groups^41^. Additionally, peaks at 1625, 1370, and 1278 cm^−1^ correspond to stretching of –C=O bonds^39^, stretching of –C–N bonds, and bending vibration of –N–H bonds^42^.

The spectrum of the hydrogel presents very notable changes in the position and intensity of the typical bands of the non-modified gelatin, in addition to the observation of new signals. Typical bands of hydrogel formation were observed at 1520–1646 cm^−1^ for the NH amino bond and the -OH band (3200–3500 cm^−1^)^43^. At 1543 cm^−1^ an intense band is observed due to the formation of C–N bond for the union of the MBA, carbonyl group of IA, and amide of acrylamide^44^. The stretching vibration was observed at 1207 cm^−1^ corresponding to –C–N-bond. The presence of these two peaks is indicative of copolymerization and crosslinking with MBA^45,46^.

Furthermore, peaks at 3648–3428 cm^−1^ refer to stretching of –OH bonds belonging to octahedral structure of the clay and adsorbed water molecules. The characteristic signals of MMT are observed at 1010 cm^−1^ and 640 cm^−1^ (stretching –Si–O–Si) and with two strain vibration bands 536 cm^−1^ (Si–O–Al) and 467 cm^−1^ (Al–OH)^47^. The spectra for the nanocomposites containing 1–5% MMT show very similar patterns. Notably some displacements and changes in intensity occur in the characteristic bands of the hydrogel and the MMT. The signals at 1750–1612 cm^−1^ demonstrate the electrostatic interaction between the carboxyl groups of the hydrogel and the cationic sites existed in MMT. In the region of 3439–3740 cm^–1^, different signals are observed that can be attributed to the interaction of amides with the surface of silane groups in MMT.

On the other hand, the XRD patterns of MMT, non-modified gelatin, grafted gelatin hydrogel, and grafted gelatin/MMT nanocomposites (1, 3, and 5% MMT) are shown in Fig. 2b. To understand the structural effects of the nanocomposites formed with the hydrogel and MMT, XRD analyzes were performed. As expected, the XRD pattern of gelatin presents a very broad peak at 2θ = 20°, representing its amorphous structure^47^. The synthesis of the gelatin-based hydrogel leads to a reduction in intensity and a broadening of the peak observed in pure gelatin, demonstrating the formation of a highly amorphous structure. The MMT diffractogram shows greater crystallinity presenting some peaks at 2θ = 19° (corresponding to plane 110), 26, and 35°. However, the incorporation of MMT into the hydrogel matrix causes these peaks to disappear and the amorphous structure conferred by the hydrogel to predominate^48^.

**Figure 3:**
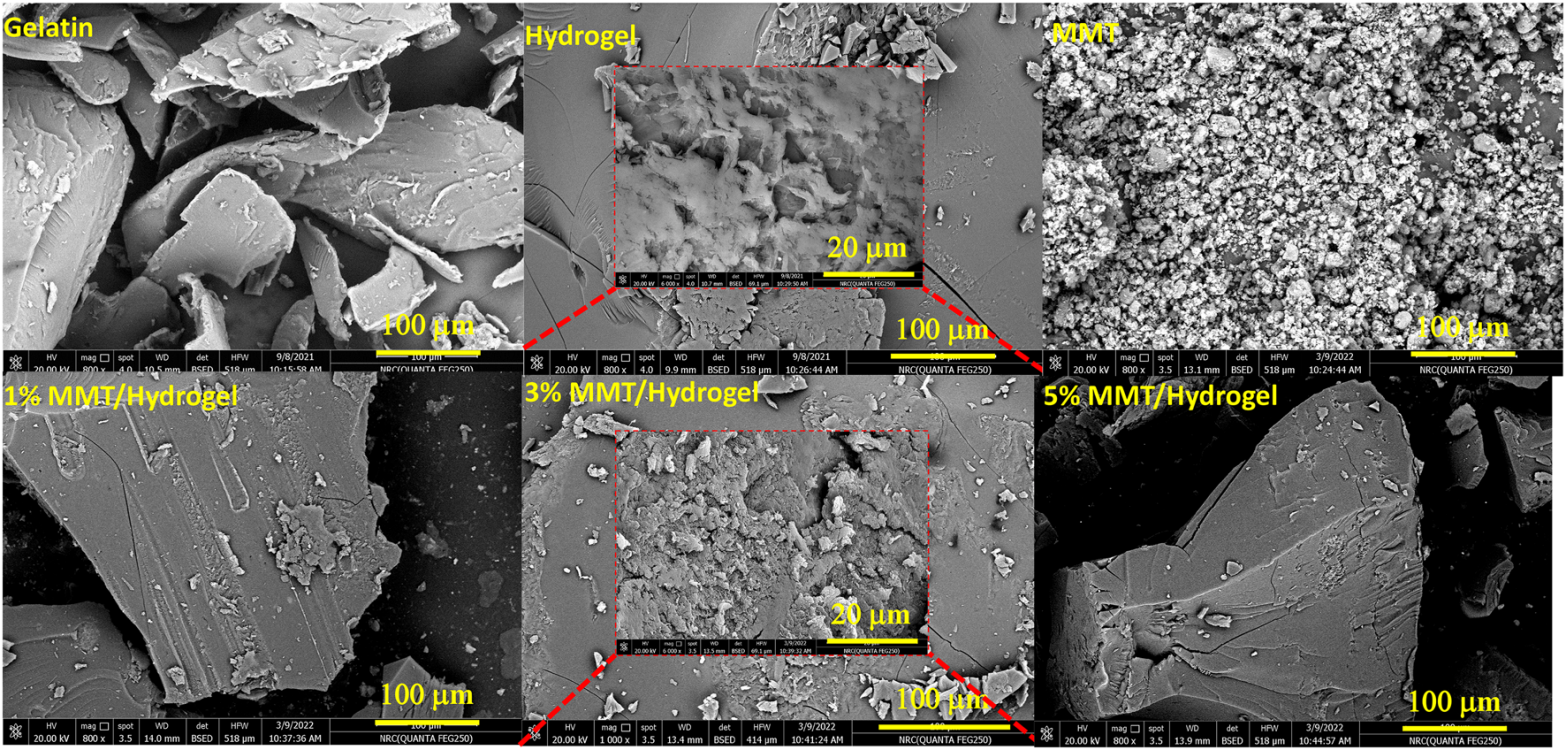
FESEM images of gelatin, modified gelatin hydrogel, MMT, and 1–5%MMT/hydrogel nanocomposites.

Moreover, the surface morphology of non-modified gelatin, modified gelatin hydrogel, MMT, and nanocomposites with MMT (1, 3, and 5%) was examined using FE-SEM technique as shown in Fig. 3, with magnification 10 μ and 20 kV.

To complement the structural and morphological behavior of the hydrogel and its nanocomposites with MMT, SEM analyses were performed. The SEM image of pure gelatin shows a rough surface with irregular arrangements. On the other hand, in the micrograph of the hydrogel, it is observed that it acquires a more porous surface that allows a greater permeability to the structure^48^. The SEM image of MMT highlights its structure composed of a mixture of randomly distributed particle aggregates and flakes. The incorporation of MMT into the polymeric matrix of the gelatin-based hydrogel results in the formation of a more compact and less porous structure at a dose of 1% MMT^49^. In a 3% dose of MMT, the nanocomposite can be seen to favor a less smooth surface, areas with a greater number of pores, and a kind of arrangement such as scaffolding that could contribute to the MG transport process and interact with a greater number of substances are identified. anionic sites along the entire internal and external surface of the nanocomposite. The surface composition of the nanocomposite was approximated by the EDX analysis, confirming the presence of the characteristic elements of Al, Si, Mg, and O. The morphology of the nanocomposite with a dose of 5% MMT is not favored in these desirable aspects for the process of adsorption of aqueous contaminants, consider that the image shows a very smooth and compact surface.

**Figure 4:**
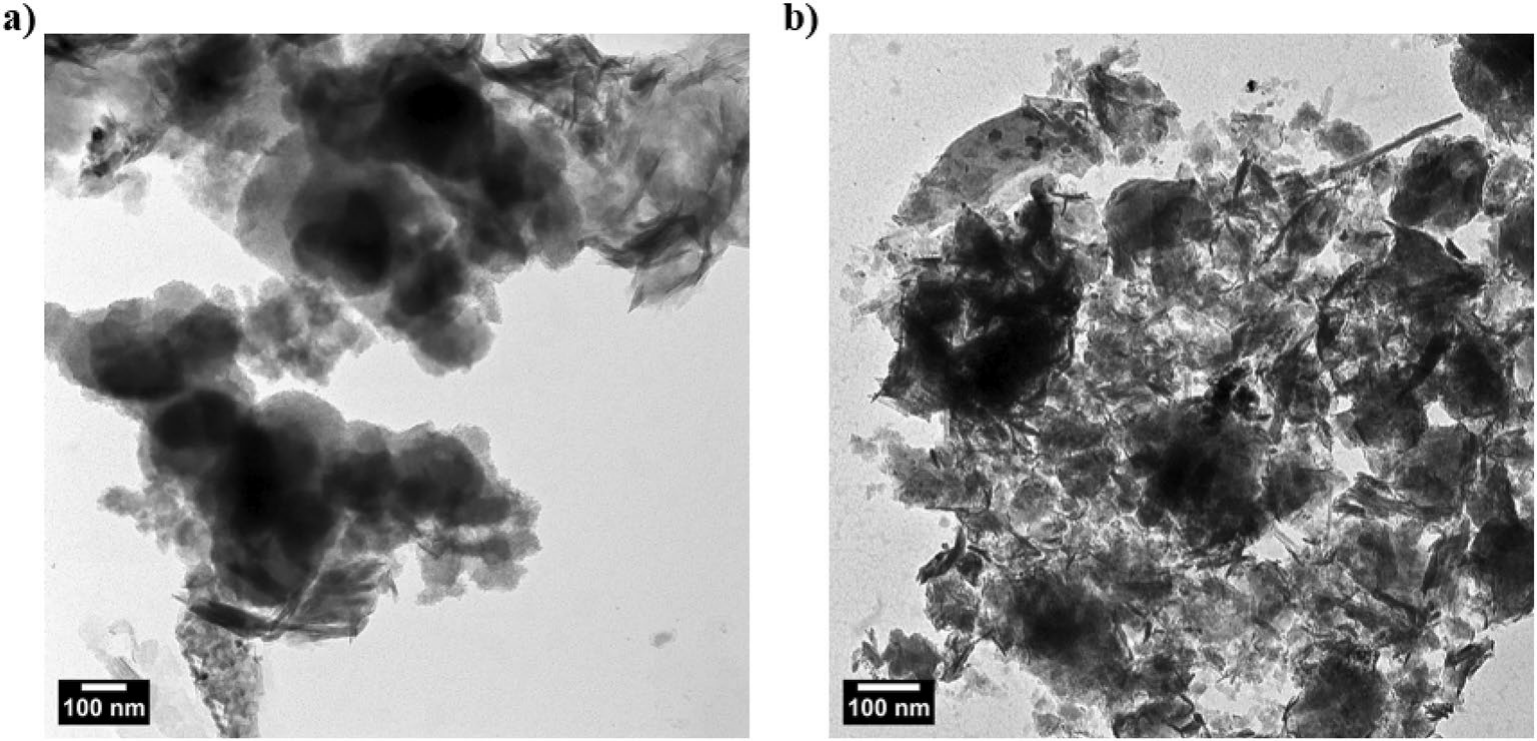
TEM images of (**a**) MMT nanoclay, and (**b**) gelatin/MMT (5%) nanocomposites.

The exfoliation of MMT nanoclay between modified gelatin hydrogels to form in situ synthesized polymer nanocomposites was confirmed by TEM technique (Fig. 4). Accordingly, MMT nanoclay (Fig. 4a) exhibited leaves-like nanosheet layers, which exfoliated homogenously between hydrogel chains. Therefore, the TEM image of polymer nanocomposite in the presence of 5% (wt/wt) (Fig. 4b) shows a good distribution of MMT nanosheets between gelatin hydrogels.

**Figure 5:**
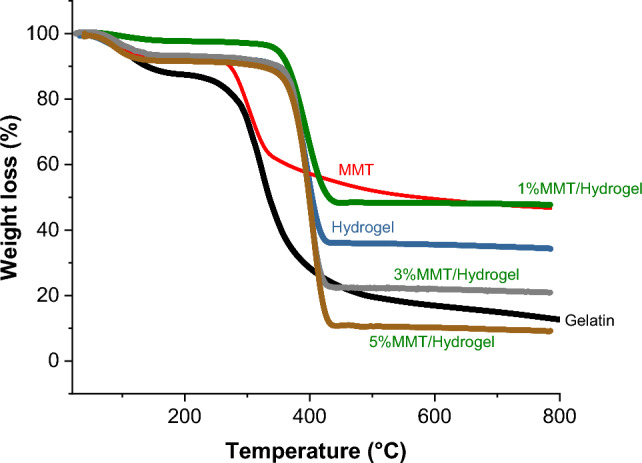
TGA thermogram of gelatin, modified gelatin hydrogel, MMT, and 1–5%MMT/hydrogel nanocomposites.

Additionally, the thermal stability of the examined samples; gelatin, gelatin hydrogel, MMT, and gelatin/MMT nanocomposites (1, 3, and 5%) was evaluated through TGA technique, and the data is shown in Fig. 5. The gelatin thermogram shows two main and characteristic trends of thermal decomposition for biopolymers. In the first, a weight loss of 13% is obtained in a range from 25 to 174 °C followed by the most pronounced loss between 250 and 500 °C attributed to a weight loss of 82%^50^.

Gelatin modification and hydrogel formation are also confirmed by the increase in thermal stability compared to pure gelatin. The hydrogel has a very similar tendency of weight loss to gelatin in the range of 25 °C to 142 °C, while the degradation of the hydrogel begins at a higher temperature (357 °C) in response to the formation of new bonds that give the hydrogel higher thermal stability. In this temperature range, a weight loss of 65% is achieved. On the other hand, MMT undergoes two stages of thermal decomposition, the first 10% weight loss is attributed to water molecules adsorbed between the pores and interlayers^50^. The weight loss between 200 °C and 400 °C can be attributed to molecules found between the interlayers of the nanoclay. Above 400°C the MTT undergoes another phase of less pronounced weight loss corresponding to the thermal degradation of the phyllosilicate of the amorphous phase^50^. The incorporation of the different doses of MMT in the hydrogel leads to a thermal behavior very similar to the trend observed for the hydrogel, where the percentages of remaining weights were 48%, 22.8% and 10.4%, for 1% MMT, 3% MMT, and 5% MMT, respectively. It is noteworthy that the 1% MMT nanocomposite exhibits the lowest weight loss.

### Adsorption process

The current study pursued to optimize the parameters of maximum adsorption capacity by preparing a high-efficient MG adsorbent based on modified gelatin /MMT nanocomposites underneath the influence of various variables, including different MMT nanoclay concentrations, initial MG dye concentration, pH of dye solution, nanocomposite dose, and adsorption contact time. The adsorption results are shown in Fig. 5.

#### Effect of MMT content

**Figure 6:**
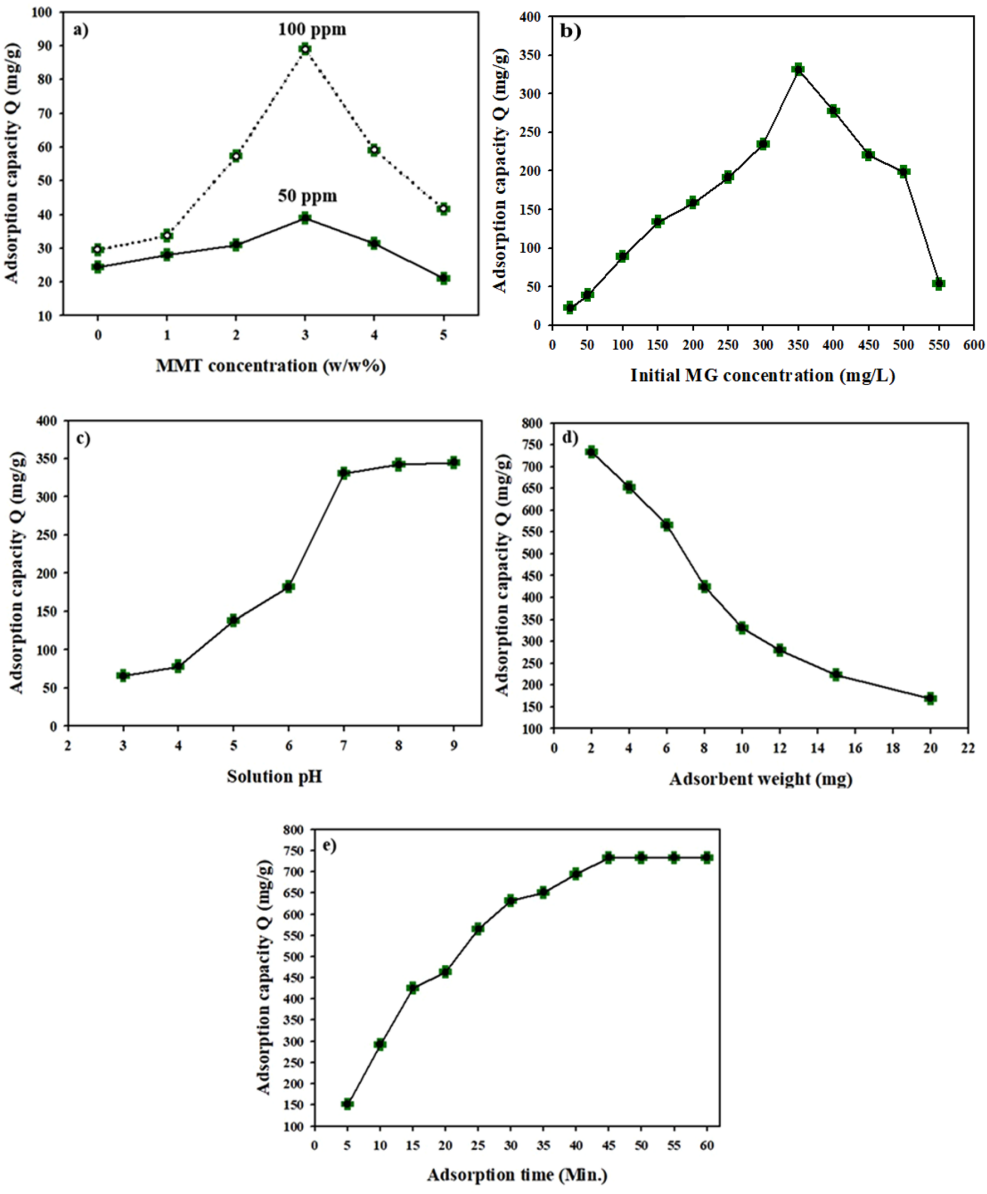
The influence of (**a**) MMT and (**b**) MG concentrations, (**c**) pH, and (**d**) polymer dose, as well as (**e**) adsorption time on MG removal by gelatin-cl-p(AAM-co-IA) /MMT nanocomposite (the standard deviation SD for three measurements for all factors is between ± 1 and ± 2).

Modified gelatin /MMT nanocomposites (1–5%) (w/w%) were compared to cross-linked gelatin hydrogel in the absence of MMT, which was used to clarify the effect of MMT concentration on MG adsorption. The effect of MMT contents (0–5%) was studied at 10 mL of initial concentrations of 50 and 150 mg/L, pH 7, and sample weight 10 mg at 30 °C for 60 min, and the data is exhibited in Fig. 6a. The results demonstrated a significant increase in dye adsorption capacity by increasing MMT ratio from 0 to 3%, with the highest value of 38.9 (mg/g) and 69.3 (mg/g) for 50 mg/L and 100 mg/L, respectively, which could be attributed to the increased surface area, and hence an increase in the number of active adsorption sites. Followed by a decline in the rejection capacity by growing up the MMT concentrations, as a result of the agglomeration of the MMT particles on the nanocomposite surface. Thus, 3% of the MMT ratio was chosen in the next experiments.

#### Effect of initial concentration of MG dye

The dye concentration effect on MG removal using gelatin-*cl*-poly (AAm-*co*-IA) (3% MMT) nanocomposites was examined at a temperature of 30 °C, different MG concentrations: 25–550 mg/L, pH 7, 10 mg adsorbent, 10 mL dye solution, and immersion time of 60 min as shown in Fig. 6b. It was found that the polymer nanocomposite adsorbent had a sufficient number of active sites, which, due to an increase in MG concentration, enhanced its adsorption capacity. Moreover, the rise in MG concentration facilitated the transfer and interaction of adsorbate and adsorbents, resulting in increased adsorption capabilities. For example, the adsorption capacity increased from 22.5 mg/g at 25 mg/L to 331.1 mg/g at 350 mg/L because of forming MG monolayer on nanocomposites^’^ surface, which is caused by the adsorption of large amounts of dye over the active sites. Subsequently, the adsorption of MG dye decreased with increasing MG concentration (above 350 mg/L), reaching 53.6 mg/g at 550 mg/L due to complete saturation for all the active sites on the 3% MMT surface. So, the MG concentration of 350 mg/L was chosen for the next study.

#### Effect of solution pH

The pH of the adsorption solution is the most essential parameter for adsorbing contaminants from water, as it has the greatest impact on the process. To identify the ideal pH value for this process, the pH influence on elimination of MG dye was investigated using 10 mg of gelatin nanocomposite in 10 mL of dye (350 mg/L) over a pH range of 3 to 9 at a temperature of 30°C within 60 min. Figure 6c shows that the rejection efficiency improved from 65.9 mg/g at pH 3 to its highest value of 345 mg/g at pH 9. Adsorption capacity increased as a result of an increase in the negatively charged groups and a decrease in H^+^ in the aqueous solution. This causes electrostatic attraction between the adsorbent surface and MG dye. At pH ˂ 5, the adsorption efficiency decreased, which may be because H^+^ was competing for surface adsorption with MG dye molecules, as well as the fact that most carboxylic groups are protonated at lower pH values; this causes repulsion among the protonated adsorbent’s surface and the positive charges on MG dye molecules. Hence, pH 9 was chosen for the next study.

#### Effect of polymer dose

Adsorption behaviour is also affected by the adsorbent dose (Fig. 6d). The dosage of gelatin nanocomposite was adjusted between 2 and 20 mg at pH 9 in 350 mg/L (10 mL) of MG solution for 60 min. The adsorption capability of adsorbent declined with increasing dosage, and maximal adsorption capacity (733 mg/g) was attained at a lower (2 mg) polymer concentration. In general, increasing the adsorbent dose reduces adsorption ability because more active sites are inaccessible to the adsorbate. This is because greater doses may promote adsorbent agglomeration and a reduction in the active adsorption sites.

#### Effect of adsorption time

The effect of the adsorption contact time of gelatin nanocomposite was studied using 2 mg of adsorbent in 10 mL of 350 mg/L MG dye, and the solution pH was adjusted to 9 during the time (5–60 min) as illustrated in Fig. 6e. The data exhibited two stages for the removal process, such as quick adsorption and gradual equilibrium.

In the first rapid adsorption stage, the adsorption capacity increased quickly with the increase in the contact time from 5 min (151.5 mg/g) to reach a maximum adsorption value of 733 (mg/g) at 45 min thanks to the intensive interaction among MG and the adsorption surface sites, which are progressively occupied.

After that, the adsorption capacity remains constant with time increase, therefore, the adsorption equilibrium was established after 45 min.

### Adsorption isotherm

The adsorption isotherm demonstrates the relationship among the examined adsorbent and adsorbate surface at equilibrium. In this study, the adsorption isotherm study was investigated for the prepared gelatin nanocomposite at optimum conditions: pH 9, 350 mg/L of MG dye (10 mL), and 2 mg of polymer within 45 min using Langmuir, Freundlich, and Temkin isotherm models.

The Langmuir model is the most widely used form of isotherm in the study of organic dye adsorption. The assumption of uniform adsorption on the adsorbent surface supports this model. As a result, the Langmuir isotherm is applied to explain the monolayer adsorption process that occurs at identified active sites. The linear Langmuir form can be expressed as the following Eq. (3)^51^.

3 $$ \frac{1}{{{\text{Q}}_{{\text{e}}} }} = \frac{1}{{{\text{Q}}_{\max } }} + \frac{1}{{{\text{Q}}_{\max } {\text{bC}}_{{\text{e}}} }} $$

where Q_e_ and Q_max_ refer to an equilibrium and maximum adsorption capacity (mg/g), respectively. While b (L/mg) is the Langmuir constant.

While the Freundlich isotherm model is used to describe heterogeneous surfaces. This model is used to describe multisite intermolecular interactions between ions that are adsorbed to active site neighbors, resulting in multilayer adsorption. The Freundlich isotherm model's linear form is described by the following Eq. (4)^52,53^.:

4 $$ {\text{In Q}}_{{\text{e}}} = {\text{ In K}}_{{\text{F}}} + \frac{1}{{\text{n}}}{\text{ ln C}}_{{\text{e}}} $$

where K_F_ (mg/g) and n are Freundlich constants for adsorption capacity and adsorption intensity, respectively.

**Figure 7:**
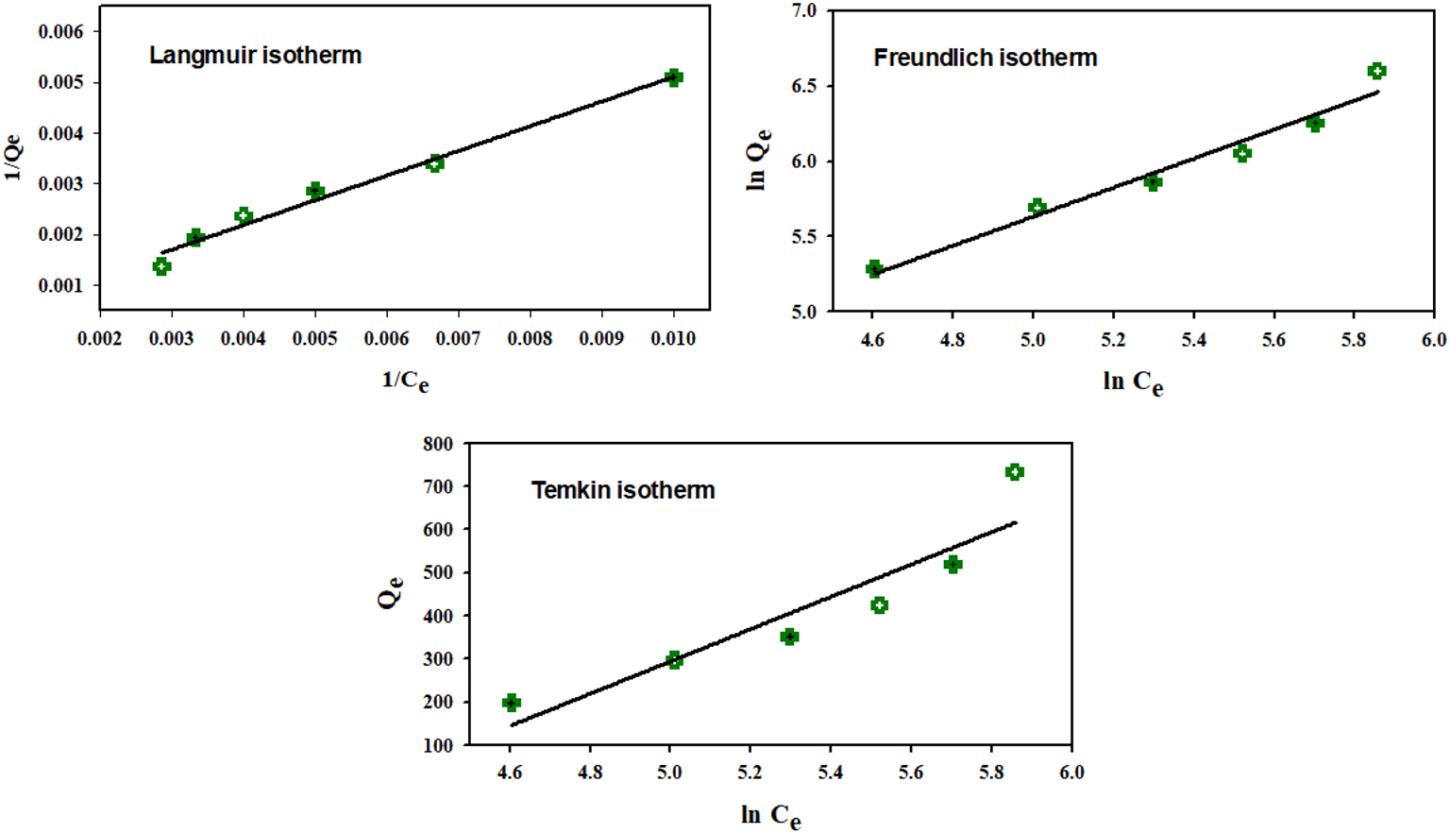
Fitting curves of linear adsorption isotherms with Langmuir model, Freundlich model, and Temkin model for removal of MG dye using modified gelatin nanocomposite.

Moreover, the multi-layer chemisorption process is the basis of the Temkin model. The Temkin isotherm model accounts for the adsorbent-adsorbate interaction through the declined adsorption heat against a heterogeneous coverage surface (Eq. (5))^21,54,55^.

5 $$ {\text{Q}}_{{\text{e}}} = \upbeta\,\, {\text{ln K}}_{{\text{t}}} + \upbeta\,\, {\text{ln C}}_{{\text{e}}} $$

where β is the constant of heat removal (= *RT/b*), b is the heat of adsorption constant (J/mol), R is the universal gas constant (8.314 J/mol/K), and T is the equilibrium temperature (273.15 K), and K_t_ is the equilibrium binding constant of the Temkin isotherm (L/g). Figure 7 and Table 1 exhibited the obtained findings, which illustrated MG removal from an aqueous solution with the synthesized nanocomposite, they were compatible with Langmuir model because it has a higher R^2^ (0.9827), which referred to the creation of homogenous MG monolayer. Additionally, Q_max_ was determined as 950.5 mg/g, while 1/n was calculated as 0.964, which confirmed a favorable adsorption process of dye molecules onto the prepared modified gelatin/MMT nanocomposite surface.

**Table 1:** Linear isotherm parameters for MG dye adsorption onto modified gelatin nanocomposites.

Langmuir model	Freundlich model	Temkin model
Q_max_ = 950.5 mg/g b = 0.0022 L/mg R^2^ (0.9827)	K_F_ = 2.26 mg/g 1/n = 0.964 R^2^ (0.9647)	β = 375.16 K_t_ = 1.3 L/g R^2^ (0.8575)

In the literature, several nanocomposite-based adsorbents were previously reported for MG dye removal from an aqueous solution; however, the prepared modified gelatin nanocomposite in this study showed significant removal than others previously reported. For example, in Table 2, there is a comparison between Q_max_ of the modified gelatin nanocomposite (950.5 mg/g) and other reported nanocomposite adsorbents for the elimination of MG dye molecules from their aqueous solutions.

**Table 2:** A comparison of Q_max_ of the prepared nanocomposite with other previously reported adsorbents for MG dye molecules.

Reported adsorbents	Q_max_ (mg/g)	References
Unsaturated polyester Ce(IV) phosphate	1.01	^56^
XG/psyllium	3.2	^57^
Poly(MMA)/GO/Fe_3_O_4_	3.5	^58^
Water nut modified carbon	47.7	^59^
Poly(AAM)-*g*-Ch/Fe_2_O_3_	86.9	^60^
CMC-*g*-poly(AAM)/MMT	172.4	^15^
Treated ginger waste	188.6	^61^
St-*g*-poly(AAM)/GO/hydroxyapatite	297.0	^62^
Ch/MMT	322.6	^63^
Ch/poly(AA)/bentonite	454.6	^64^
XG-*g*-poly(AA-co-AAM)/Fe_3_O_4_	497.2	^65^
XG-*g*-poly(VI)/SiO_2_	588.2	^20^
Alg/poly (AA)/graphite	628.9	^66^
Modified GG/SiO_2_	781.3	^67^
XG-*g*-poly(VI)/MMT	909.1	^21^
Gelatin-*cl*-poly(AAm-co-IA)/MMT	950.5	Present work

### Adsorption kinetic studies

The time of MG adsorption could affect its removal performance using the prepared modified gelatin nanocomposite. The kinetic adsorption study of capturing MG dye investigated within the adsorption relation among removal time and adsorbed MG dye molecules onto nanocomposite surface. Indeed, the removal process includes MG movement from solution to adsorbent’s interface, and then MG diffusion to reach the inner active sites^68,69^. The kinetic performance of MG adsorption using gelatin-*cl*-p(AAM-*co*-IA)/MMT nanocomposite was investigated using pseudo-first order, pseudo-second order, second order, and Weber-Morris intraparticle diffusion models.

**Figure 8:**
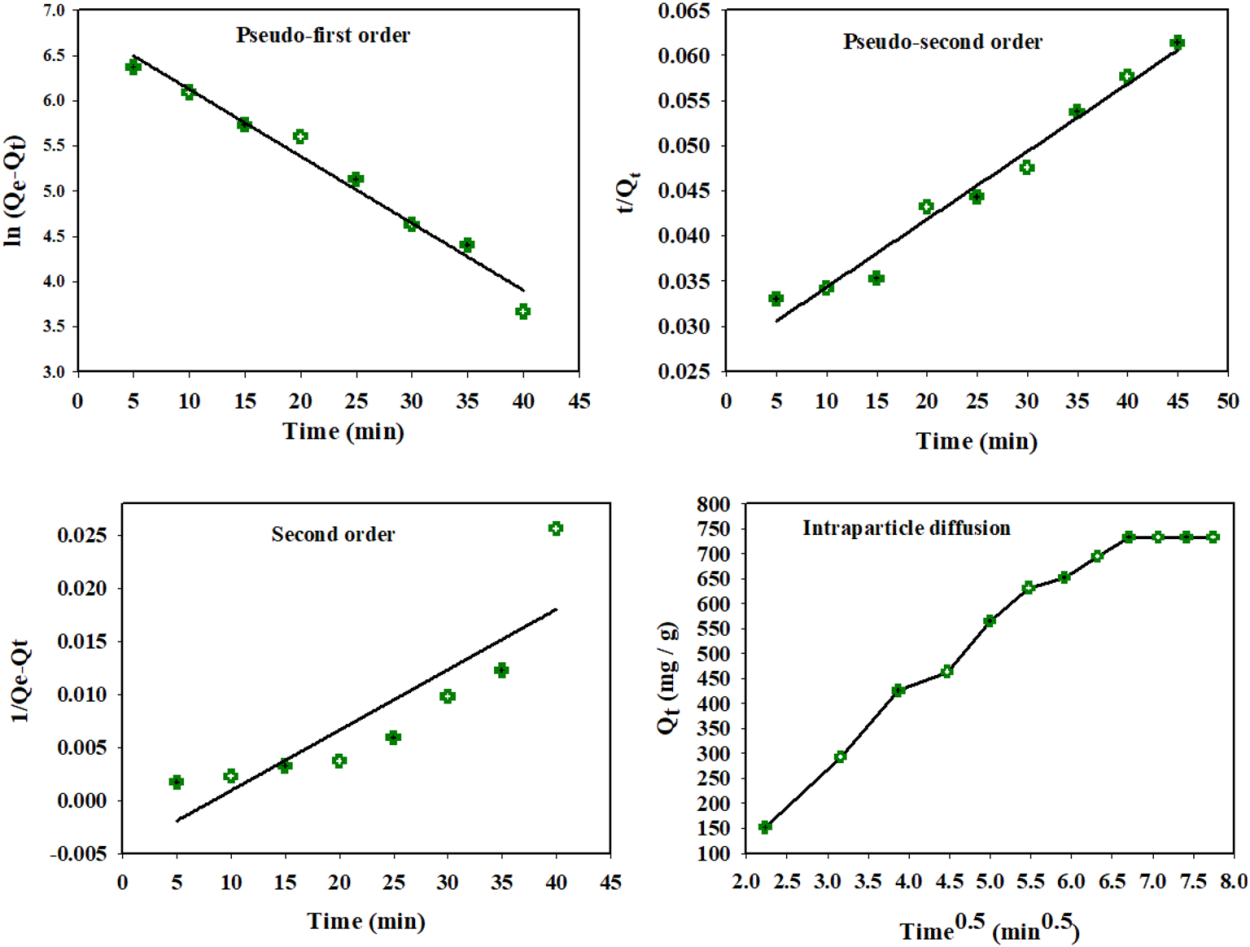
Fitting curves of linear pseudo-first-order, pseudo-second-order, second-order, and Weber–Morris models for MG rejection using modified gelatin nanocomposite.

Their linear equation forms can be expressed by the following Eqs. (6–9), respectively^70–73^, and their findings are illustrated in Fig. 8 and Table 3.

6 $$ {\text{ln }}({\text{Q}}_{{\text{e}}} - {\text{Q}}_{{\text{t}}} ) \, = {\text{ ln Q}}_{{\text{e}}} - {\text{K}}_{{1}} {\text{t}} $$

7 $$ \frac{{\text{t}}}{{{\text{Q}}_{{\text{t}}} }} = \frac{1}{{{\text{K}}_{{2}} {\text{Q}}_{{\text{e}}}^{2} }} + \frac{{\text{t}}}{{{\text{Q}}_{{\text{e}}} }} $$

8 $$ \frac{1}{{{\text{Q}}_{{\text{e}}} - {\text{Q}}_{{\text{t}}} }} = \frac{1}{{{\text{Q}}_{{\text{e}}} }} + {\text{K}}_{3} {\text{t}} $$

9 $$ {\text{Q}}_{{\text{t}}} = {\text{ K}}_{{4}} {\text{t}}^{{0.{5}}} + {\text{ C}} $$

where Q_t_ (mg/g) is the adsorption capacity of adsorbents at different contact time (t); K_1_ (1/min), K_2_ (g/mg. min), K_3_ (g/mg. min), K_4_ (mg/g. min^0.5^) represent the rate constant of pseudo-first order, pseudo-second order, second order, and Weber-Morris models, respectively. While C refers to the thickness of nanocomposite surface’s boundary layer. The data shown illustrated that pseudo-first order has higher R^2^ (0.9744), and Q_e_, which was calculated from the same model, was very close to Q_max_ that was recorded from Langmuir isotherm model (Table 1). Therefore, pseudo-first order is the most promising model for the removal of MG dye using the prepared gelatin nanocomposite.

**Table 3:** The parameters of kinetic adsorption models for the rejection of MG dye using gelatin nanocomposite.

Pseudo-first order	Pseudo-second order	Second order	Intraparticle diffusion
K_1_ = 0.0741 min^−1^ Q_e_ = 960.45 mg/g R^2^ (0.9744)	K_2_ = 9.3 × 10^–8^ g/mg min Q_e_ = 1250 mg/g R^2^ (0.9742)	K_3_ = 0.001 g/mg.min Q_e_ = 212.7 mg/g R^2^ (0.7574)	K_4_ = 108.14 mg/g min^0.5^ C = 133

Moreover, Fig. 8 exhibited that Weber-Morris intraparticle diffusion model includes two straight sections, corresponding to multi-steps adsorption process. The first linear section resulted from the immigration of MG from bulk solution to boundary external layer of adsorbent to be adsorbed on its surface via strong electrostatic and hydrogen bonding interactions. Meanwhile second straight-line exhibits intraparticle diffusion process of MG molecules through the inner nanocomposite surface pores.

On the other hand, C = 133, which refers to boundary layer thickness. As a result, data confirmed that Weber-Morris model is a rate-limiting step for the rejection of dye molecules. According to the findings, the pseudo-first order and Weber–Morris intraparticle diffusion models participated together for the adsorption mechanism of MG dye on the surface of modified gelatin/MMT nanocomposite.

### Chemical elucidation of MG loaded- gelatin/MMT nanocomposites

**Figure 9:**
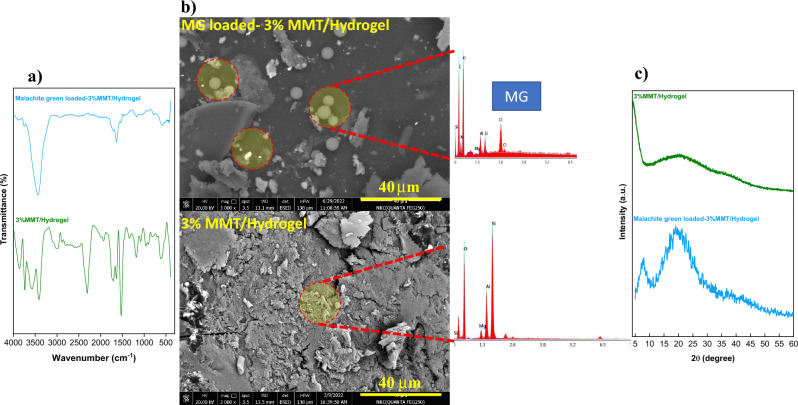
Physicochemical characterization of 3% MMT nanocomposite and MG loaded-3% MMT nanocomposite: FTIR spectrum (**a**), SEM images-EDX (**b**) and XRD pattern (**c**).

The elucidation of the chemical structure of the adsorbent before and after MG adsorption may be used to suggest a reasonable mechanism for MG adsorption by gelatin-*cl*-p(AAM-*co*-IA)/MMT nanocomposite (Fig. 9). The structure of MG adsorbed nanocomposite was studied compared with unloaded nanocomposite using different techniques: FTIR, XRD, FE-SEM, and EDX.

The elucidated MG adsorption mechanism is supported by the characterization and study of the nanocomposite before and after loading the cationic dye. In the FTIR spectrum for 3% MMT/hydrogel and MG loaded- 3% MMT/hydrogel, we can observe that the suppression of some characteristic signals of the nanocomposite, discussed above, occurred which indicates the adsorption effect of MG at different active sites, and different types of chemical interactions that promote their effective removal. SEM images highlighted the formation of MG clusters or crystals adsorbed on the surface of the nanocomposite. EDX analysis confirms the chemical composition of these zones and shows the content of chlorine atoms coming from MG dye molecules. Additionally, XRD pattern indicates that the adsorption of MG confers a more amorphous structure to the nanocomposite, a peak at 2θ = 5.2° characteristic for MG dye is also observed.

### Adsorption MG dye mechanism

**Figure 10:**
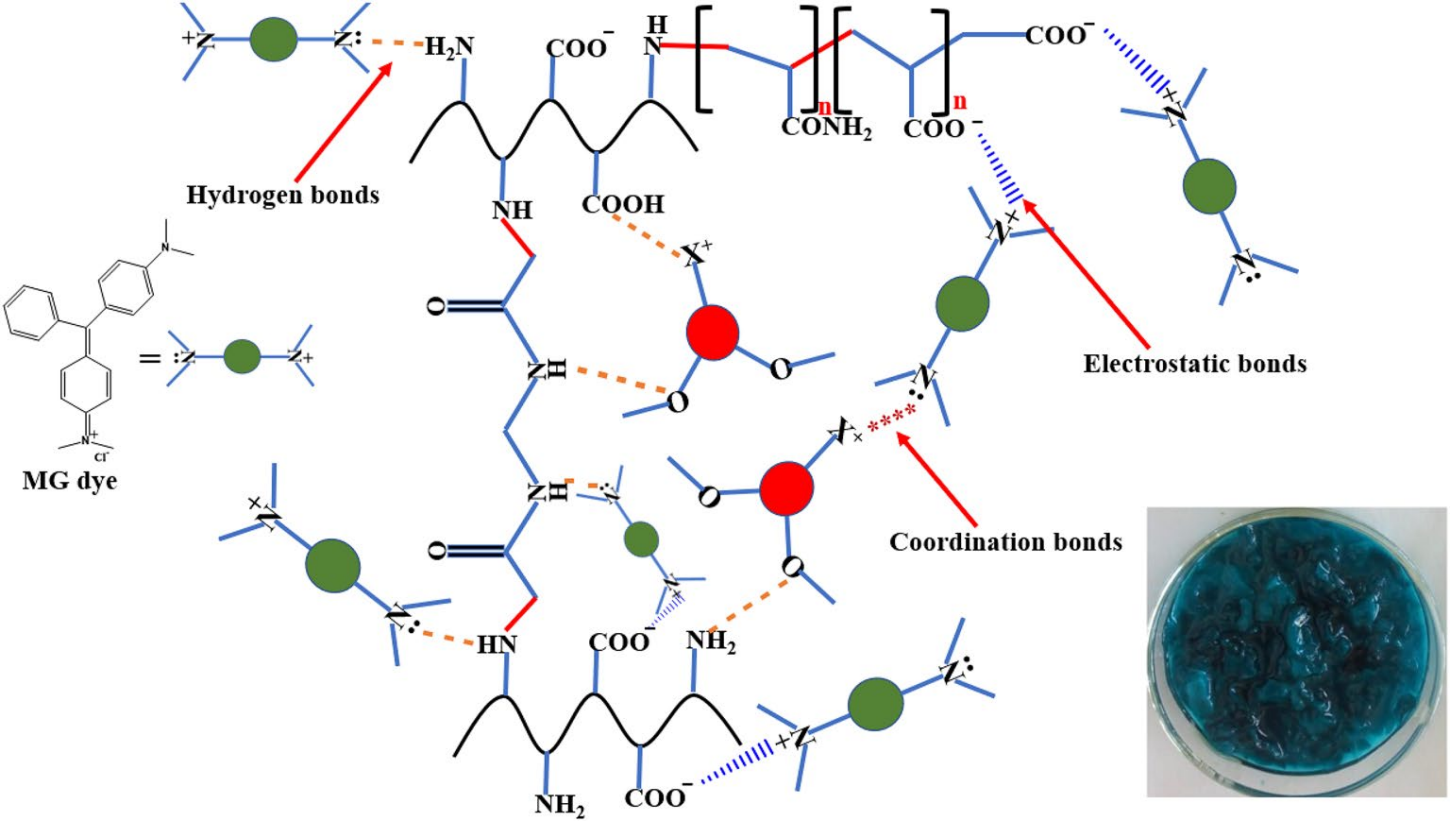
Schematic illustration of the proposed adsorption mechanism of MG dye with modified gelatin nanocomposite.

A possible adsorption mechanism between MG dye molecules and the adsorbent nanocomposite is shown in Fig. 10. Accordingly, the interaction mechanism here consists mainly of: electrostatic forces, coordination bonding, and hydrogen bonding interactions. The electrostatic interactions formed between cationic quaternized amino groups on MG dye surface and the negative groups onto the nanocomposite surface. While the coordination interactions were created among metal ions of MMT nanoclay and the lone pairs on tertiary amino groups onto the dye surface as well as H-bonding interaction bonds.

### Regeneration study

**Figure 11:**
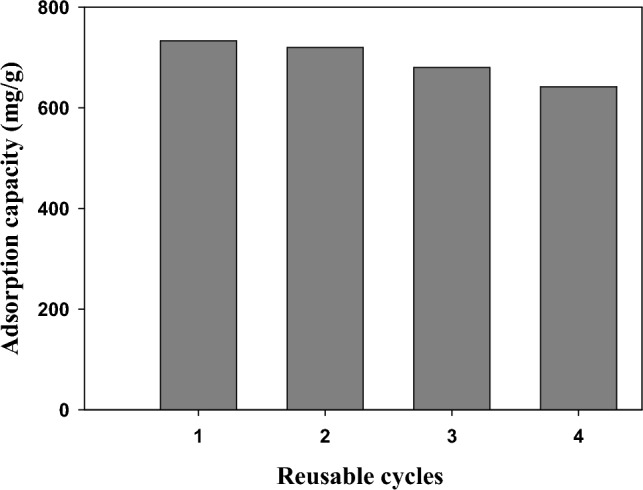
Schematic diagram of regeneration study within four consecutive reusable cycles.

Adsorbent regeneration is one of the most important methods for determining the most efficient adsorbent for wastewater treatment applications. Real-world applications account for the management of pollutant-loaded adsorbents. Indeed, the reusability of pollutant-loaded adsorbent materials is crucial for both the economic and environmental sectors^66,68,74^. The regeneration of the MG-loaded nanocomposite was done through four reusable cycles and the findings exhibited that adsorption capacity was determined as 733 mg/g (1st cycle), 719.9 mg/g (2nd cycle), 680.1 mg/g (3rd cycle), and 641.8 mg/g after 4th cycle (Fig. 11). This test confirmed the good recyclability of the prepared modified nanocomposite for MG dye rejection.

## Conclusion

In the present work, gelatin-*cl*-p(AAM-*co*-IA)/MMT nanocomposite was successfully synthesized with free-radical polymerization approach as a potential adsorbent for rejecting MG dye from an aqueous solution. The developed adsorbent was elucidated using various analytical instrumentation: FTIR, XRD, TGA, TEM, FE-SEM, and EDX techniques. The data confirmed that MMT nanoclays were mixed homogenously among grafted gelatin chains, thereby reinforcing the adsorption and thermal properties of the polymeric nanocomposites, comparing with non-modified gelatin and modified gelatin hydrogel. The adsorption MG dye process was influenced by various parameters. According to the adsorption process, the nanocomposite adsorbent was well fitted with Langmuir isotherm model (R^2^ value of 0.9827), with maximum adsorption efficiency of 950.5 mg/g. Meanwhile, the adsorption kinetics results were compatible with pseudo-first order and intraparticle diffusion kinetic models.

The adsorbents were successfully regenerated in four adsorption/desorption cycles, and the findings indicated good recyclability for the prepared modified gelatin nanocomposite. The adsorption process between MG dye molecules and adsorbent nanocomposite was dominated by electrostatic force, coordination bonding, and hydrogen bonding interactions. Overall, the fabricated nanocomposite is one of the most promising low-cost and highly efficient adsorbent for water purification.

## Data Availability

The data will be available from the corresponding author upon request.
